# Biofilm forming abilities of *Salmonella *are correlated with persistence in fish meal- and feed factories

**DOI:** 10.1186/1746-6148-5-20

**Published:** 2009-05-27

**Authors:** Lene K Vestby, Trond Møretrø, Solveig Langsrud, Even Heir, Live L Nesse

**Affiliations:** 1 National Veterinary Institute, PO Box 750 Sentrum, N-0106, Oslo, Norway; 2 Nofima mat, Osloveien 1, N-1430, Aas, Norway

## Abstract

**Background:**

Feed contaminated with *Salmonella *spp. constitutes a risk of *Salmonella *infections in animals, and subsequently in the consumers of animal products. *Salmonella *are occasionally isolated from the feed factory environment and some clones of *Salmonella *persist in the factory environment for several years. One hypothesis is that biofilm formation facilitates persistence by protecting bacteria against environmental stress, e.g. disinfection. The aim of this study was to investigate the biofilm forming potential of *Salmonella *strains from feed- and fishmeal factories. The study included 111 *Salmonella *strains isolated from Norwegian feed and fish meal factories in the period 1991–2006 of serovar Agona, serovar Montevideo, serovar Senftenberg and serovar Typhimurium.

**Results:**

Significant differences were found between serovars regarding the abilities to form biofilm on polystyrene (microtiter plate assay) and in the air-liquid interface of nutrient broth (pellicle assay). Strains of serovar Agona and serovar Montevideo were good biofilm producers. In Norwegian factories, clones of these serovars have been observed to persist for several years. Most serovar Senftenberg clones appear to persist for a shorter period, and strains of this serovar were medium biofilm producers in our test systems. Strains of the serovar Typhimurium were relatively poor biofilm producers. *Salmonella *ser. Typhimurium clones have not been observed to persist even though this serovar is resident in Norwegian wild life. When classifying strains according to persistence or presumed non-persistence, persistent strains produced more biofilm than presumed non-persisting strains.

**Conclusion:**

The results indicate a correlation between persistence and biofilm formation which suggests that biofilm forming ability may be an important factor for persistence of *Salmonella *in the factory environment.

## Background

*Salmonella *spp. are important enteropathogenic pathogens, which may cause disease in humans, animals and birds. The bacteria are mainly transmitted via the faecal-oral route, and salmonellosis is one of the most common and widely distributed foodborne diseases in humans. According to the WHO, millions of human cases are reported worldwide every year, and the disease results in thousands of deaths . The occurrence of *Salmonella *in feed and feed ingredients is a well-recognized problem worldwide [1-5]. This constitutes a considerable risk of *Salmonella *infection in animals, and subsequently in the consumers of animal products [6]. Therefore, large resources are put into the fight against *Salmonella *in feed- and fishmeal factories. Still, some clones seem to be able to persist in the factory environment for several years [7]. Earlier studies have showed that these clones are not particularly resistant to disinfection or air-drying at surfaces [8]. Because biofilm protects bacteria against environmental stress, e.g. disinfection [9], one hypothesis is that biofilm formation facilitates persistence. This has been shown for *Listeria monocytogenes *[10], but to the best of our knowledge, a similar linkage between biofilm formation and persistence in the environment has not been shown for *Salmonella*.

Bacterial biofilms are defined as microbial sessile communities that are attached to a substance, to an interface or to each other [11]. In the biofilm, the cells are embedded in a self produced matrix which may act as chemical and mechanical protection against the surroundings [12,13]. Bacteria in biofilms are known to be more resistant to disinfectants and drying [14-16]. Recent studies have shown that *Salmonella *are capable of forming biofilm on different contact surfaces like glass, polymer, steel [17-20] along with organic surfaces like parsley [21]. *Salmonella *ser. Typhimurium has also been shown to form biofilm (pellicle) on the liquid-air interface of nutrient broth [15,22]. Many disinfectants which are commonly used in feed production environment were shown to have low bactericidal activity against strains from Norwegian feed- and fishmeal factories in biofilm [14].

The aim of the present study was to investigate whether the biofilm forming abilities of *Salmonella *strains at room temperature may contribute to persistence in the factory environment. The serovar Agona, serovar Montevideo and serovar Senftenberg have for many years been the ones most frequently isolated from Norwegian factories, but are rarely found in humans and animals [7,23]. On the other hand, *Salmonella *ser. Typhimurium, which is endemic in Norwegian wildlife [24], is seldom found in the factories. Therefore, the biofilm producing abilities of these serovars were compared.

## Methods

### Bacterial strains and culture conditions

A total of 116 *S. enterica *strains of serovar Agona (n = 39), serovar Montevideo (n = 30), serovar Senftenberg (n = 34) and serovar Typhimurium (n = 13) were used in this study, including 111 strains from Norwegian feed and fish meal factories, isolated in the years 1991–2006 [7,23], as well as the National reference strains of serovar Agona (FHBA266), serovar Montevideo (FHBA46), serovar Senftenberg (FHBA87) and serovar Typhimurium strains ATCC 14028 and ATCC 2700720D (LT2). The factories that submitted *Salmonella *to this study all have internal *Salmonella *control, based on the HACCP system. Samples were taken once or twice a month throughout the production period, from products as well as from critical control points in the environment such as filters, drains, product contact surfaces and other surfaces. Some factories also collected samples from raw materials. Environmental samples were collected as dust, incrustations and swabs. All isolations were carried out at different private or official laboratories and verified at the National Reference Laboratory for *Salmonella *in Feed and Food at the National Veterinary Institute.

Forty-four of the strains were characterised by pulsed field gel electrophoresis (PFGE) as described earlier [7,23]. If strains with the same PFGE profile had been isolated from the same factory over a time period of at least one year, the strain that was last isolated was classified as persistent. A strain was classified as presumed non-persistent if no other strain with the same PFGE-profile had been isolated from the same factory. Based on these criteria, the study included seven persistent strains (four serovar Agona, three serovar Montevideo) and 14 presumed non-persistent strains (two serovar Agona, three serovar Montevideo, three serovar Senftenberg, six serovar Typhimurium). All of these strains originated from environmental or product samples, and not from raw materials.

Nine strains from the collection were used to test the effect of prolonged incubation in microtiter plates; the serovar Typhimurium ATCC strains 14028 and LT2 (700720D), the national reference strains of serovar Agona, serovar Montevideo and serovar Senftenberg and one randomly selected factory strain of each of the same serovars. All the factory strains (n = 111) were included in the screening on liquid, whereas the screening in microtiter plates included 61 factory strains (19 serovar Agona strains, 12 serovar Montevideo strains, 21 serovar Senftenberg strains and nine serovar Typhimurium strains). The selection criteria for screening in microtiter plates were to represent a diversity of PFGE profiles, to include isolates from as many feed and fish meal factories and years as possible in the study material.

All strains were stored at -80°C in Brain Heart Infusion broth (BHI; Difco, BD, NJ, USA) supplemented with 15% glycerine (Merck KGaA, Darmstadt, Germany) and recovered on bloodagar (sheepblood) at 37.0 ± 1.0°C overnight. The bacterial cultures were then transferred into Luria Bertani broth (LB; Merck KGaA) and incubated statically overnight at 37.0 ± 1.0°C. Luria Bertani without NaCl (LB ^wo^/NaCl; bacto-tryptone 10 g/L, yeast extract 5 g/L) was used as test broth in the biofilm assays [25].

### Biofilm on polystyrene

The assay was based on the method described by Woodward and associates [20]. In short; overnight cultures were diluted in LB ^wo^/NaCl to OD_595 _= 0.2, and 30 μL of this suspension were transferred to each well in 96 wells polystyrene microtiter plates (Nunc, Nuncleon, Roskilde, Denmark) containing 100 μL LB ^wo^/NaCl (three parallels of each strain), and the microtiter plates were incubated statically for two days, at 20.0 ± 1.0°C. When testing the effect of prolonged incubation, the plates were incubated statically at 20.0 ± 1.0°C for one, two, three and four days. After incubation, OD_595 _were measured before the plates were gently washed once with sterile distilled water (SDW). The plates were dried in room temperature before addition of 130 μL 1% crystal violet. After 30 minutes incubation in room temperature, the plates were washed three times with SDW before addition of 130 μL ethanol:acetone (70:30 w:w) and incubation for 10 minutes in room temperature. OD_595 _were measured after the bound dye was dissolved using ethanol:acetone. For each strain, the result was calculated by subtracting the median OD_595 _of the three parallels of the control (test broth only) from the median OD_595 _of the three parallels of sample.

### Biofilm in liquid- air interface

To study biofilm formation in liquid, i.e. pellicle formation at the liquid-air interface, 4.5 mL LB ^wo^/NaCl was inoculated with 0.5 mL of an overnight culture, and incubated statically at 20.0 ± 1.0°C for eight days. The strains were visually examined every day and categorized according to pellicle formation or not [26].

### Statistics

Statistical analyses were performed using the software JMP (SAS Institute Inc.version 5.0.1a, Cary, NC, USA) and Minitab (release 14.2, Minitab Inc., PA. USA). Log_10_-transformed values were used when necessary.

## Results

### Biofilm on polystyrene

**Figure 1:**
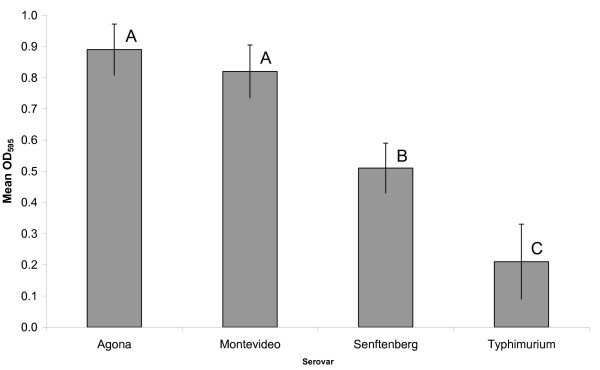
**Biofilm formation in microtiterplates**. Mean amount of biofilm formation of different *Salmonella *serovars in microtiter plates at 20°C represented by OD_595_. Bars represent standard error of the mean. Levels not connected by the same letter are significantly different (p < 0.05).

In the screening of 61 factory strains in microtiter plates, OD_595 _values were found to range from 0 to 1.46. Statistically significant differences between serovars were observed (One-way ANOVA, p < 0.01) (Figure 1). Highest OD_595 _values were observed in serovar Agona and serovar Montevideo strains (mean 0.89 and 0.82, respectively), medium OD_595 _values in the serovar Senftenberg strains (mean 0.51), and lowest OD_595 _values in serovar Typhimurium strains (mean 0.21). The persistent strains had higher OD_595 _(mean 1.01) than the presumed non-persistent strains (mean 0.44) (Students' t test, p < 0.05). As persistent strains were only found within serovar Agona and serovar Montevideo, a comparison was also performed using the persistent and presumed non-persistent strains of these serovars only. Also in this comparison, the persistent strains displayed a higher OD_595 _than the presumed non-persistent strains (mean OD_595 _1.01 vs 0.69, Students' t test, p = 0.05). By incubating the subset of nine strains for a prolonged time (two, three and four days), increased OD_595 _values after three and four days were only observed for the serovar Senftenberg strains.

### Pellicle formation in liquid

**Figure 2:**
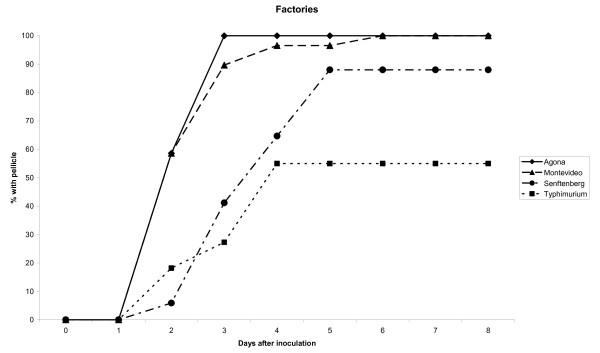
**Pellicle formation in liquid-air interface**. Percentage of strains within each serovar with a pellicle in standing liquid at different incubation times.

All strains were tested for their ability to form biofilm in standing liquid, i.e. formation of a pellicle at the liquid-air interface. Pellicle formation was found to vary between serovars (Chi square p < 0.01) (Figure 2). High frequencies of pellicle formation were found among serovar Agona, serovar Montevideo and serovar Senftenberg strains (100, 100 and 88%, respectively), while only 55% of the serovar Typhimurium strains formed a pellicle. Pellicle producers had a higher mean OD_595 _in microtiter plates (mean 0.68, STD = 0.42) than strains without pellicle (mean = 0.40, STD = 0.50) (Students' t test, p < 0.05). The rate of pellicle formation also varied between the serovars tested (Students' t test, p < 0.05) (Figure 2). Strains of serovar Agona and serovar Montevideo were rapid pellicle producers, whereas serovar Senftenberg and serovar Typhimurium were slow pellicle producers. The number of days needed to form a pellicle was negatively correlated to OD_595 _observed in microtiter plates (linear fit, p < 0.01), i.e. the higher the OD_595_, the shorter time was needed to form a pellicle. The mean number of days needed to form a pellicle was lower in the persistent strains than in the presumed non-persistent strains, both when including all the defined strains (2.4 vs 3.5, Students' t test, p < 0.05), and when testing the serovar Agona and serovar Montevideo strains only (2.4 vs 3.6, Students' t test, p < 0.05).

## Discussion

Several clones of serovar Agona and serovar Montevideo have persisted in some Norwegian feed- and fishmeal factories over a number of years [7,23]. In our study, these serovars were in general the good biofilm producers in microtiter plates. Furthermore, most strains of these serovars rapidly formed a biofilm (pellicle) at the air-liquid interface. *Salmonella *ser. Senftenberg has also been repeatedly isolated from feed- and fishmeal factories, but pulsed-field gel electrophoresis studies have shown that these strains display a relative large number of different profiles [23]. This may indicate that many serovar Senftenberg clones enter the factories and may persist for a while, but probably not as long as serovar Agona and serovar Montevideo. However, serovar Senftenberg is the serovar most commonly isolated from imported feed raw materials [1] and this may contribute the high prevalence in the factory samples. The serovar Senftenberg strains studied were medium biofilm producers in the microtiter plate assay. Although most of the serovar Senftenberg strains did form a pellicle, the rate was slow. The serovar Typhimurium is known to be endemic in Norwegian wild life [24] and is probably present in the surrounding environment of most factories. Still, this serovar is rarely isolated from factories, and has not been observed to persist. The serovar Typhimurium strains tested produced little biofilm in microtiter plates. As many as 45% of the strains did not produce a pellicle, and the remaining strains were slow pellicle producers. In summary, the strongly persistent serovar Agona and serovar Montevideo were good biofilm producers, the medium persistent serovar Senftenberg was a medium biofilm producer and the non-persistent serovar Typhimurium displayed the weakest biofilm forming abilities in our test systems. These observations support the hypothesis that the biofilm forming ability is an important factor for persistence of *Salmonella *strains in the factory environment.

This hypothesis is further supported by the results showing that the persistent strains clearly were better biofilm producers in both test systems than the presumed non-persistent strains. To exclude the possibility that serovar may be a confounding variable, we also compared biofilm production by persistent and presumed non-persistent strains of serovar Agona and serovar Montevideo only. Also in this comparison, the persistent strains were better biofilm producers than the presumed non-persistent strains, indicating that the observed correlation between persistence and biofilm production is not due to a confounding effect of serovar. In our study, the factories had for over a number of years sent us *Salmonella *isolates that they have found through their internal control routines. This provided a good knowledge of the *Salmonella *situation in the different factories over time. In addition, we chose relatively strict criteria for classifying strains as persistent and non-persistent, compared to similar studies performed with *Listeria monocytogenes *[27,28]. The classification of strains is therefore believed to be as reliable as possible.

Consequently, all our results favour the hypothesis that biofilm forming abilities at room temperature are linked to persistence of *Salmonella *in fish meal and feed production environments. To our knowledge, such a linkage has not been reported previously for *Salmonella*. However, similar results has been reported for *Listeria monocytogenes *[10], where differences in biofilm formation was detected between persistent and non-persistent strains of from bulk milk samples [27], and from poultry plants and an ice cream plant [28]. Even though biofilm formation has been shown to be important for persistence, it is clearly not the only contributing factor. Being a good biofilm producer gives an advantage, but opportunities for biofilm formation must also be present, e.g. failure in HACCP control routines that enables *Salmonella *to establish in the environment and form biofilm. Other characteristics of the bacteria may also contribute to persistence.

All assays in the present study were performed at 20°C. According to representatives from the feed- and fishmeal factories involved in this study, the temperatures in different parts of the factories may vary from 5 to 40°C, although around 20°C is most common. In this study, biofilm formation on polystyrene and on the air-liquid interphase was assessed. This may not be the most common surfaces in the factory environment. However, in studies with a limited number of strains we found a correlation between biofilm formation of *Salmonella *on polystyrene and stainless steel at room temperature (results not shown). Thus the biofilm characteristics determined in this study should be of relevance regarding the biofilm forming potential of the *Salmonella *strains in the feed factory environment.

Several factors may contribute to the amount of biofilm observed in the microtiter plate assay at a given length of incubation, e.g. ability to adhere to the surface, the rate of biofilm formation, detachment of parts of the biofilm. In the present study, a correlation between the results from the two assays was observed, indicating that the OD_595 _in microtiter plates after two days was influenced by the production rate. However, when testing a subset of stains in the microtiter plate assay over prolonged incubation periods, only the serovar Senftenberg strains displayed a significant increase in OD_595 _values from day two to day four. Consequently, the production rate was probably the major limiting factor for the serovar Senftenberg strains in the microtiter plate assay when the incubation time was only two days. This was supported by the results from the pellicle assay, where the majority of the serovar Senftenberg strains did produce a pellicle given enough time. On the other hand, prolonged incubation did not substantially increase the OD_595 _in the microtiter plate assay or the number of pellicles produced by the serovar Typhimurium strains. Therefore, other factors than rate are probably more limiting within this serovar.

## Conclusion

In conclusion, the present study showed significant differences between serovars regarding biofilm formation on polystyrene and the liquid-air interface. The results indicate that the ability to form biofilm is important for the bacteria's persistence in feed factory environments.

## Authors' contributions

LKV was responsible for the study design, performing all the screening experiments, analysis of the data, and preparation of the manuscript, and LLN participated in all these parts. TM and SL were responsible for establishing the microtiterplate method. All authors contributed to the study design and revision of the draft manuscript. All authors have read, edited and approved the final manuscript.
